# Differentiation of benign nevoid rests and metastatic melanoma in sentinel lymph node biopsy

**DOI:** 10.1093/jscr/rjad036

**Published:** 2023-02-09

**Authors:** Joseph Phillipos, Afaq Khan, Neil Jayasuriya

**Affiliations:** General Surgery, La Trobe Regional Hospital, Traralgon, Australia; Anatomical Pathology, Dorovitch Pathology, Heidelberg, Australia; General Surgery, La Trobe Regional Hospital, Traralgon, Australia

**Keywords:** sentinel lymph node, benign nevoid rests, melanoma

## Abstract

Patients with cutaneous melanoma routinely undergo sentinel lymph node (SLN) biopsy. If this first lymph node is clear, the entire lymph node basin is very likely to be free from the metastatic disease. Lymph node analysis is therefore of great importance with respect to prognostication and further management. Various cell types, including benign nevoid rests, can mimic metastatic melanomatous cells in the SLN. There is no standardized method to differentiate naevoid rests from metastatic melanoma. Diagnosis is based on cell location, morphology and multiple immunohistochemical techniques, with no single test being completely diagnostic. We present a patient with Lentigo Maligna melanoma, who was found to have benign nevoid rests on SLN biopsy, and discuss the diagnostic tests and considerations in differentiating benign nevoid rests from metastatic melanoma.

## INTRODUCTION

The sentinel lymph node (SLN) biopsy, first described by Morton [1], is based on the hypothesis that tumour cells metastasize to the first nodes receiving lymphatic drainage before involving other lymph node tiers. If this first node, known as the SLN, is clear, the entire lymph node basin is highly likely to be free from tumour cells [1, 2]. In patients with cutaneous melanoma with risk of regional nodal metastasis, SLN biopsy is routinely performed. Identification of malignant cells in lymph nodes can prove to be challenging because multiple cell types, including benign nevoid rests, can mimic malignant melanomatous cells. There is no definitive test to diagnose metastatic melanoma, and there is an overlap in cytomorphology and immunohistochemical (IHC) staining patterns between metastatic melanoma and nevoid cells. Differentiation has to made in SLN biopsy, as it has implications for prognostication and management. Diagnosis entails a multi-faceted approach and may include a second histopathologist’s opinion.

## CASE REPORT

We present a 53-year-old male truck driver, who presented with an ill-defined, palpable brown nodular lesion on the posterior aspect of his left forearm, measuring ~12 × 10 mm. He was a non-smoker and denied spending significant amounts of time in the sun. Duration of the presence of the lesion was unknown.

**Figure 1:**
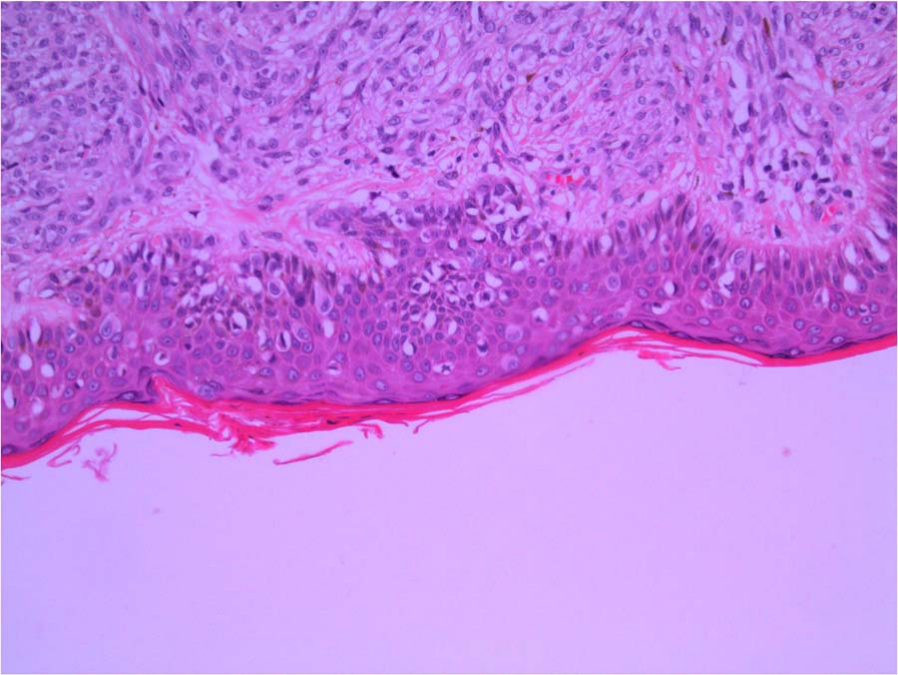
Wide local excision of primary lesion demonstrating pagetoid spread in epidermis and infiltration into reticular dermis; H&E × 200 magnification.

Excisional biopsy of the primary lesion was performed by his general practitioner which confirmed the diagnosis of Lentigo Maligna melanoma. Breslow thickness was 1.9 mm, and the deep margin demonstrated an invasive component to 2 mm, equating to Clark 4 invasion. Junctional melanocytes showed moderate to marked atypia, with sheets of melanocytes in the dermis (Fig. 1). There was no ulceration or lymphovascular invasion. This was followed by wide local re-excision. Adjacent epidermis showed a mild increase in atypical melanocytes, with no contiguous proliferation. No residual *in situ* or invasive malignancy was detected. He underwent a staging computed tomography (CT) that did not identify distant metastases.

**Figure 2:**
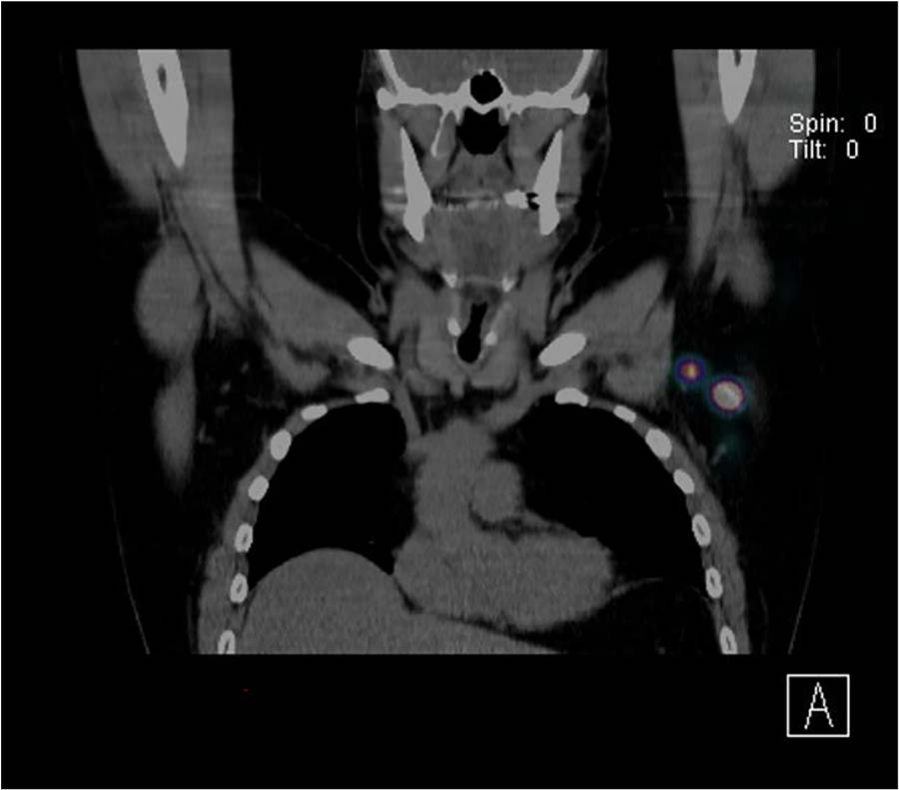
CT SPECT image identifying two SLNs in the left axilla.

Further wide local excision was performed with margins of 2 cm to deep fascia and keystone flap reconstruction. SLN biopsy was carried out, with dual localization of SLN with Patent Blue V and radio nucleoid tracer. The preop CT single-photon-emission computed tomography (SPECT) imaging showed a focus of tracer activity in two SLNs in the left axilla (Fig. 2). The two lymph nodes were harvested with standard technique.

**Figure 3:**
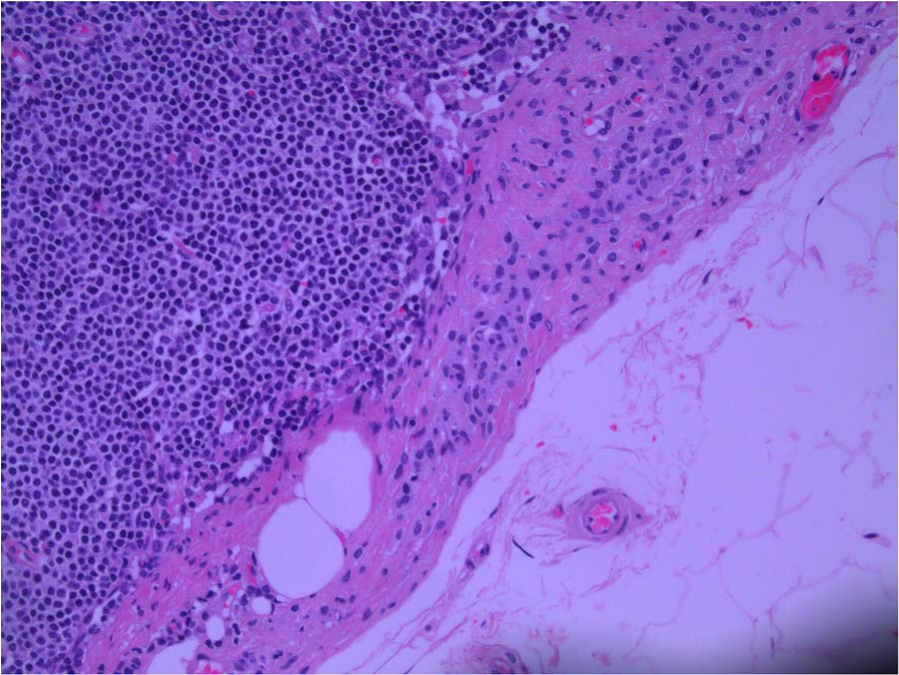
Lymph node; nevoid melanocytes in subcapsular region; H&E × 200 magnification.

**Figure 4:**
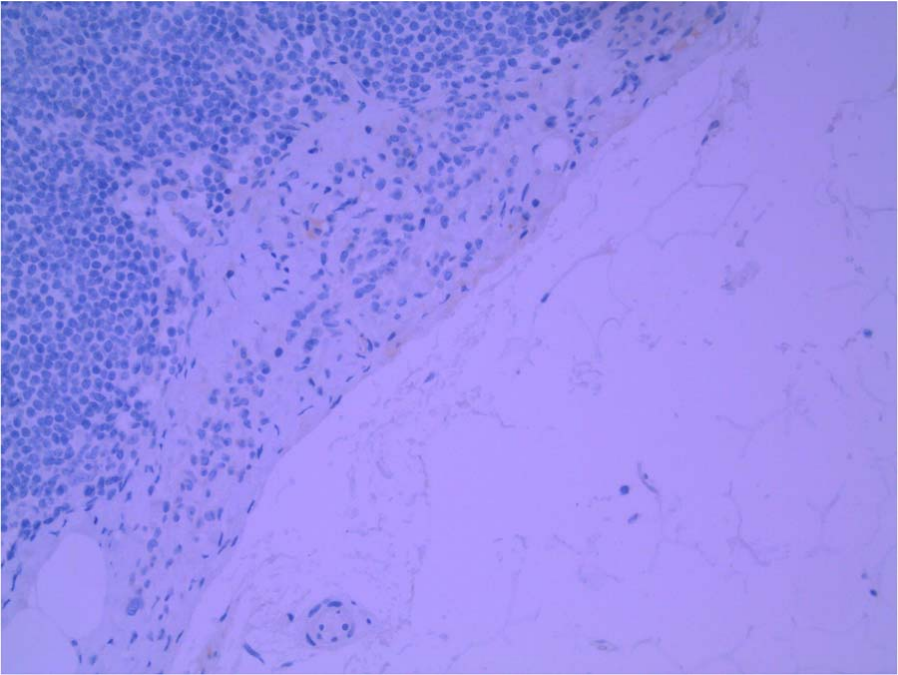
Lymph node with IHC using HMB-45 stain, not staining subcapsular region melanocytic cells; × 200 magnification.

**Figure 5:**
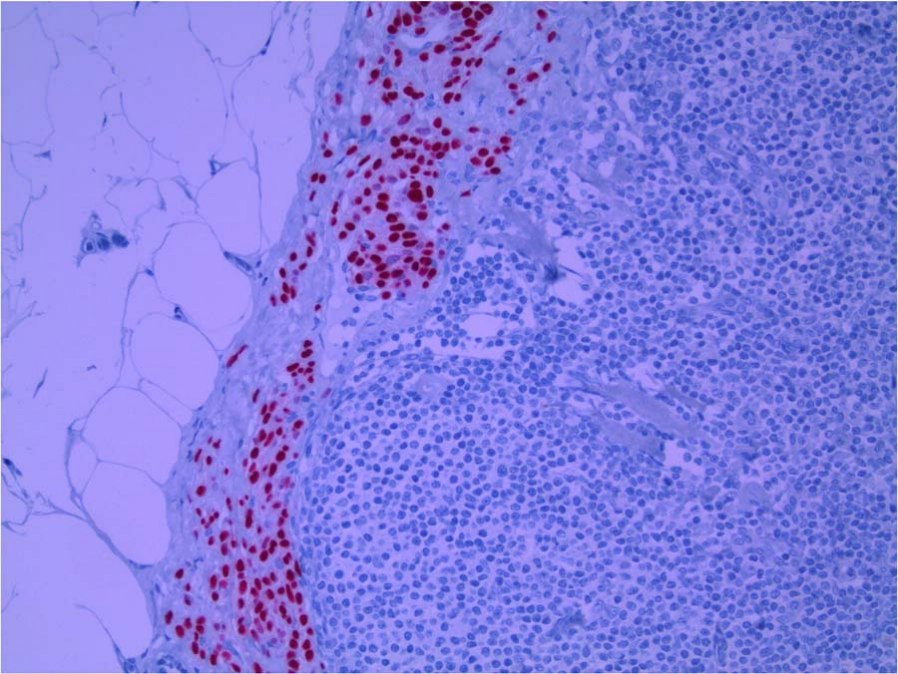
Lymph node with IHC using Sox 10 stain, staining melanocytic cells (benign or malignant) in subcapsular region; × 200 magnification.

**Figure 6:**
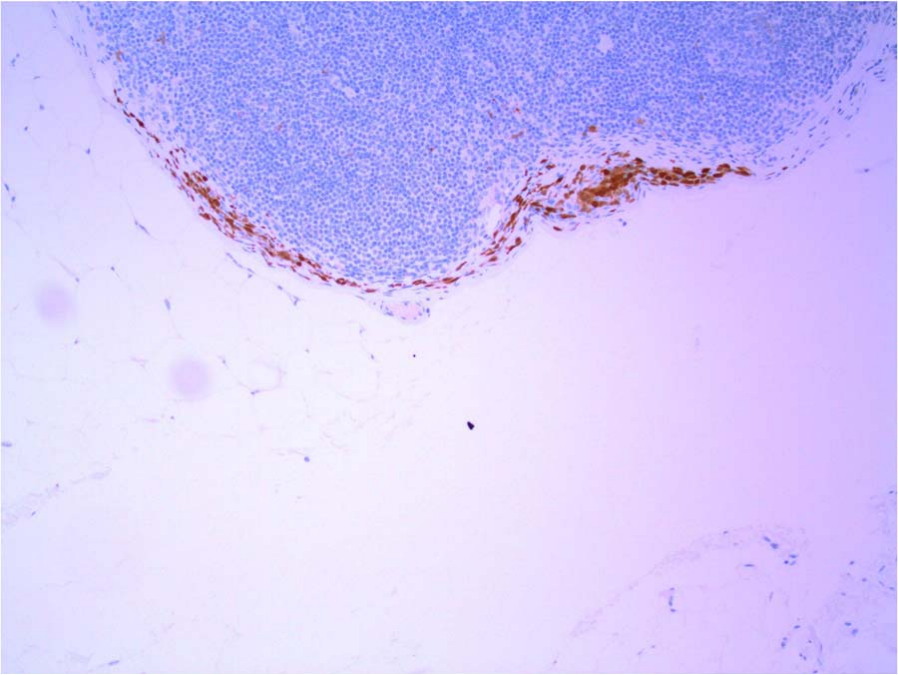
Lymph node with IHC using p16 stain, staining nevoid melanocytes; × 100 magnification.

Lymph node specimens were sent for haematoxylin and eosin (H&E) staining (Fig. 3) and IHC analysis. The sections showed mild reactive changes and benign intracapsular nevoid rests. IHC analysis showed that these cells were negative for HMB-45 (Fig. 4) and were positive for both Sox10 and p16 (Figs 5 and 6). Consensus at the Multidisciplinary Team Meeting was that the appearance was in keeping with benign naevoid rests as opposed to melanoma deposits, given the location of the cells, morphology and immunohistochemistry. Surveillance was recommended.

## DISCUSSION

Naevoid rests are benign embryological remnants of melanocytic cells. Patients with benign naevus cell aggregates in SLN biopsy have a prognosis similar to patients with negative lymph nodes and do not require further intervention [3]. Patients with lymph nodes positive for metastatic melanoma may require adjuvant systemic therapy and/or surgical resection [4, 5]. Similarities in cytomorphology and architecture as well as IHC markers can make differentiation difficult [3, 6].

Currently, the gold standard for diagnosis of melanoma involves tissue morphology and histopathology of the primary lesion. Histopathological diagnosis incorporates architectural, cytologic and host response features, with atypical melanocytes and architectural disorder required. The Australian Clinical Practice Guidelines state that SLN biopsy should be considered in patients with cutaneous melanoma >1 mm in thickness or >0.8 mm with high risk pathological features [7]. SLN biopsy is a multi-step procedure. The first step is lymphoscintigraphy in which a radioactive tracer is injected. Gamma camera imaging is used preoperatively and a gamma probe aids in the correct identification of the SLN/s intraoperatively. Localized SLN/s are then removed and are assessed for the presence of metastatic disease. Intranodal melanocytes usually indicate metastatic disease [8], however, benign naevoid rests can occasionally present in lymph nodes.

Lymphatic drainage enters the node at the level of the subcapsular sinus; thus, most nodal metastasis are found in the subcapsular region [9]. Naevoid rests are usually found within the nodal fibrous capsule and nodal trabeculae, as was the case for the current patient. Naevoid rests can present in the subcapsular region, complicating the interpretation of lymph node status.

H&E staining is the gold standard for lymph node evaluation, and IHC staining is used to improve sensitivity and specificity [10–13]. Similar to the staining of the primary lesion, there is an overlap between the commonly used markers, and no single marker has 100% specificity or sensitivity.

Several IHC stains are in use in the pathological interpretation of primary lesions and SLN biopsies. The antibody HMB-45 reacts with most melanomas. In a primary lesion, HMB-45 is immunoreactive with intraepidermal and superficial dermal components of benign nevi [14]. In a lymph node, however, benign nevoid cells are negative for HMB-45 and appear bland. A loss of HMB-45 expression has been reported in 20% of melanocytic metastasis, illustrating the need for further diagnostic testing [14]. Sox10 is a nuclear transcription factor that stains benign and malignant melanocytic cells. It indicates the extent of melanocytic spread but does not differentiate between benign nevi and metastatic melanoma [15]. Immunostaining for the tumour suppressor gene p16 differentiates between benign naevi and melanocytic metastases in the SLN. One study demonstrated positive nuclear and cytoplasmic p16 staining in all nevi (dermal and lymph node) and the absence of nuclear p16 staining in all but one melanoma metastasis [14]. No single stain is completely sensitive for melanocytic metastases, and a confident diagnosis relies upon cell location, morphology and multiple IHC techniques. SLN biopsy in the current patient demonstrated intracapsular melanocytic cells which stained negative for HMB-45, and positive for Sox10 and p16, in keeping with benign nevoid rests as opposed to metastatic melanoma.

Differentiation between melanoma and benign nevus is highly important for patient outcomes and resource allocation, however, similarities in cytomorphology and IHC techniques for both diagnosis of the primary lesion and interpretation of SLNs can make diagnosis and staging problematic. Differentiating between benign naevoid rests and metastatic melanomatous cells requires multiple pathological techniques and consideration of cell morphology and location.

## CONFLICT OF INTEREST STATEMENT

None declared.

## FUNDING

None.

## References

[1] Morton DL , Wen D-R, Wong JH, Economou JS, Cagle LA, Storm FK, et al. Technical details of intraoperative lymphatic mapping for early stage melanoma. Arch Surg 1992;127:392–9. 1558490 10.1001/archsurg.1992.01420040034005

[2] Krag DN , Meijer SJ, Weaver DL, Loggie BW, Harlow SP, Tanabe KK, et al. Minimal-access surgery for staging of malignant melanoma. Arch Surg 1995;130:654–8. 7539252 10.1001/archsurg.1995.01430060092018

[3] Davis J , Patil J, Aydin N, Mishra A, Misra S. Capsular nevus versus metastatic malignant melanoma–a diagnostic dilemma. Int J Surg Case Rep 2016;29:20–4. 27810606 10.1016/j.ijscr.2016.10.040 PMC5094157

[4] Stebbens W , Garibyan L, Sober A. Sentinel lymph node biopsy and melanoma: 2010 update part II. J Am Acad Dermatol 2010;62:737–48. 20398811 10.1016/j.jaad.2009.11.696

[5] Wong SL , Balch CM, Hurley P, Agarwala SS, Akhurst TJ, Cochran A, et al. Sentinel lymph node biopsy for melanoma: American Society of Clinical Oncology and Society of Surgical Oncology joint clinical practice guideline. Ann Surg Oncol 2012;19:3313–24. 22766987 10.1245/s10434-012-2475-3

[6] Cohen LM . Lentigo maligna and lentigo maligna melanoma. J Am Acad Dermatol 1995;33:923–36. 7490362 10.1016/0190-9622(95)90282-1

[7] Rapport F , Smith AL, Cust AE, Mann GJ, Watts CG, Gyorki DE, et al. Identifying challenges to implementation of clinical practice guidelines for sentinel lymph node biopsy in patients with melanoma in Australia: protocol paper for a mixed methods study. BMJ Open 2020;10:e032636. 10.1136/bmjopen-2019-032636 PMC7050375 32111612

[8] Carson KF , Wen D-R, Li P-X, Lana AM-A, Bailly C, Morton DL, et al. Nodal nevi and cutaneous melanomas. Am J Surg Pathol 1996;20:834–40. 8669531 10.1097/00000478-199607000-00006

[9] Murray C , Leong W, McCready D, Ghazarian D. Histopathological patterns of melanoma metastases in sentinel lymph nodes. J Clin Pathol 2004;57:64–7. 14693838 10.1136/jcp.57.1.64 PMC1770187

[10] Gibbs JF , Huang PP, Zhang PJ, Kraybill WG, Cheney R. Accuracy of pathologic techniques for the diagnosis of metastatic melanoma in sentinel lymph nodes. Ann Surg Oncol 1999;6:699–704. 10560857 10.1007/s10434-999-0691-2

[11] Messina JL , Glass LF, Cruse CW, Berman C, Ku NK, Reintgen DS. Pathologic examination of the sentinel lymph node in malignant melanoma. Am J Surg Pathol 1999;23:686–90. 10366151 10.1097/00000478-199906000-00008

[12] Prieto VG . Sentinel lymph nodes in cutaneous melanoma: handling, examination, and clinical repercussion. Arch Pathol Lab Med 2010;134:1764–9. 21128773 10.5858/2009-0502-RAR.1

[13] Chopra A , Sharma R, Rao UN. Pathology of melanoma. Surgical Clinics 2020;100:43–59. 31753115 10.1016/j.suc.2019.09.004

[14] Mihic-Probst D , Saremaslani P, Komminoth P, Heitz PU. Immunostaining for the tumour suppressor gene p16 product is a useful marker to differentiate melanoma metastasis from lymph-node nevus. Virchows Arch 2003;443:745–51. 14576937 10.1007/s00428-003-0897-9

[15] Willis BC , Johnson G, Wang J, Cohen C. SOX10: a useful marker for identifying metastatic melanoma in sentinel lymph nodes. Appl Immunohistochem Mol Morphol 2015;23:109–12. 25356946 10.1097/PAI.0000000000000097

